# Understanding the survivorship burden of long COVID

**DOI:** 10.1016/j.eclinm.2021.100767

**Published:** 2021-02-24

**Authors:** Fahad M. Iqbal, Kyle Lam, Viknesh Sounderajah, Sarah Elkin, Hutan Ashrafian, Ara Darzi

**Affiliations:** a Department of Surgery & Cancer, Imperial College London, St. Mary's Hospital, London, W2 1NY, UK; b Institute of Global Health Innovation, Imperial College London, South Kensington Campus, Kensington, London, SW7 2AZ, UK

Emerging evidence suggests that upwards of 20% of all SARS-CoV-2 positive individuals continue to experience chronic and debilitating symptoms, known either as ‘long COVID’ or ‘post COVID syndrome’, following the resolution of their initial infection [1]. Despite this sizeable cohort, there have been limited coordinated attempts to understand the overall survivorship burden associated with this condition.

The concept of ‘survivorship’ provides healthcare professionals, researchers and policy makers with a communal lens through which they may frame holistic interventions aimed at reducing the overall burden of living through a condition. This term encompasses the physiological, psychological, social, functional and economic impact of living with a chronic condition for an affected individual and their family members/caregivers [2]. Despite its predominant use in oncological literature, we can draw several parallels between the journey of long COVID and many cancers; both patient cohorts typically describe (i) the psychological impact of an unexpected diagnosis and duration of symptoms; (ii) a complex set of evolving physical symptoms; (iii) on-going changes in physical function; and (iv) an associated change in lifestyle, finances and interpersonal relationships [3].

These themes have been distilled into nine distinct survivorship domains: treatment complications, physical function, co-morbidities, caregivers, employment & finance, relationships & family, mental health, social functioning and self-care [3]. By focusing on these themes, we are able to characterise, evaluate, and form interventions to combat chronic conditions, such as long COVID.

The majority of existing survivorship literature centres around ‘complications’ of post-COVID syndrome [4]. There seems to be an absence in the influence of existing co-morbidities on the course of post-COVID syndrome. Further work to establish relationships between post-COVID syndrome and common comorbidities (e.g. underlying lung pathology or immunosuppression therapies) would be merited. As such, the survivorship burden associated with co-morbidities remains unclear.

EQ-5D index values, a surrogate for quality of life, were lower when compared to the country specific index population norm [5]. However, given that population changes are dynamic, initial reference values published in 2014 may poorly represent the ‘physical function’ domain. Additionally, the ‘employment and finance’ domain has been briefly described, but merits additional investigation with non-hospitalised individuals excluded during the acute infection [6].

Patient experiences of post-COVID syndrome have reported themes relating to enduring persisting symptoms, anxiety, difficulty in accessing care, and uncertainty [7]. Although qualitative literature has potential to relate to other domains, themes involving self-care, social function, relationships, and financial implications require further exploration alongside the growing body of work in the ‘mental health’ domain [8].

There is a pressing need for both breadth and longitudinal evidence in survivorship, particularly in the treatment complications, social function and self-care survivorship domains. As such, we are left with the following recommendations:

1) Healthcare professionals must not only recognise the symptomatology of post-COVID syndrome but also understand the longer-term support that patients require in the community. For policy makers, it is only through objectively quantifying the impact of this condition on both an individual and societal level are they able to lobby for appropriate funding and resources at a governmental level. Once more, parallels can be drawn from cancer survivorship in which a strong foundation of evidence has driven the development of frameworks, such as the National Cancer Survivorship Initiative, now deemed essential for the provision of personalised care.
2) There is also a responsibility for both researchers and patients in the coming months. Triallists should be urged to collaborate in order to develop ‘joined up’ research studies which are complimentary and avoid research waste. A promising first step has been undertaken by The National Institute for Health Research (NIHR) and the international long-term care policy network, who have formed dedicated steering groups to coordinate large scale international research studies [9,10]. Patient and public education is also a key consideration in this process. In spite of the malaise that has developed amongst large sections of the public, continued efforts must be made to actively recruit people with long COVID into trials.
3) Finally, public education concerning long COVID will help to dispel stigma around the condition thus reinforcing the previous two recommendations. There is a need for long COVID to be publicly recognised as a legitimate health condition, so that there will be support for allocation of public funds towards long COVID. Parallels can be drawn with mental illness where it is only relatively recently that mental illness has been commonly recognised as a genuine disease on a par with physical illness and thus resources for sufferers have been increased. Increasing awareness and reducing stigma around long COVID will also encourage recruitment of individuals to clinical trials.

## Declaration of Competing Interest

None
